# Exploration of nutritional, pharmacological, and the processing trends for valorization of finger millet (*Eleusine coracana*): A review

**DOI:** 10.1002/fsn3.3659

**Published:** 2023-09-08

**Authors:** Rhythm Kalsi, Jasleen Bhasin, Gulden Goksen, Piyush Kashyap

**Affiliations:** ^1^ Department of Food Technology and Nutrition, School of Agriculture Lovely Professional University Phagwara Punjab India; ^2^ Department of Food Technology Vocational School of Technical Sciences at Mersin Tarsus Organized Industrial Zone, Tarsus University Mersin Turkey

**Keywords:** finger millets, nutritional, pharmacological, phytochemical, processing, utilization

## Abstract

High nutrient variability and food security are the needs of the hour. Millets may be as effective as other cereal crops for dealing with severe malnutrition and increasing global population problems. Due to their physiologically active components, millets have attracted more research interest. Finger millet (FM), one of the climate‐resilient and minor cereal crop species, is well known for several health benefits, primarily attributed to its nutritional value and polyphenolic content. FM seed coat phenolics exhibit excellent anti‐diabetic, anti‐oxidant, antimicrobial, anti‐osteoporosis, wound healing, anti‐lithiatic, inhibiting collagen glycation, cross‐linking, and enzyme properties, which may serve well for the pharmacological purposes. Furthermore, the processing of FM is an important factor in its commercial use. It is necessary to invent some novel technologies to increase the productivity of FM by lowering the cost of processing and its effective utilization in the pharmaceutical and food industries. The literature presented will further explore the potential prospects of processing as well as value‐added utilization and its nutritional and pharmacological aspects in view of initiating further research in the food industry to formulate ready‐to‐eat and ready‐to‐cook products, thereby acting as future crops for sustainability.

## Abstract 2

High nutrient variability and food security are needed and millets may be as effective as other cereal crops for dealing with severe malnutrition and increasing global population problems. The literature presented will further explore the potential prospects of processing as well as value‐added utilization and its nutritional and pharmacological aspects in view of initiating further research in the food industry to formulate ready‐to‐eat and ready‐to‐cook products, thereby acting as a future crop for sustainability.

## 1 INTRODUCTION

Nutritional insecurity poses a severe threat to the world's population, primarily dependent on cereal‐based diets and lacking essential micronutrients. The nutritional quality of a community has been acknowledged as a key determinant of national growth. As hunger impedes national development, it has been observed as a worldwide issue. Adequate intake of a quality diet is advisable to address the issues of pervasive food insecurity and malnutrition. One of the potentially effective methods for enhancing food security is the cultivation of traditional and primitive food crops in particular regions, which are now replaced with non‐commercial food crops (Singh & Raghuvanshi, 2012). Together with grains, millets are one of the major sources of energy throughout Asia, Africa, and the semi‐arid tropical regions of the world and are thus regarded as “wonder crops.” These small‐seeded annual grass species belong to the family Poaceae and any of the genera *Panicum*, *Setaria*, *Echinocloa*, *Pennisetum*, and *Paspalum* in the tribe *Paniceae* and genus *Eleusine*, with a shorter life span than other cereal crops (Gaikwad et al., 2021). FAOSTAT reports that global millet production in 2016 was reported to be 30.35 million metric tons, and Indian millet production is approximately 10 million tons (Himanshu et al., 2018). These wonder crops can withstand unfavorable climatic conditions featuring droughts, frequent climate changes, and moderate soil fertility (Bommy & Maheswari, 2016) and are thus recognized as “climate compliant or resilient crops.” They are the reservoir of various nutrients, containing about 60%–75% carbohydrates, 7%–11% proteins, 1.5%–5% fats, 2%–7% crude fiber, vitamins (B9, B7, B2, B1), and minerals (calcium, iron, potassium, magnesium, and zinc), and phytochemicals (phenols, tannins, alkaloids, and saponins), which, in a nutshell, help combat nutritional deficiencies. Considering the nutritional value of millets, several millet‐based food products (bakery, weaning, fermented, snack, and health foods) can be prepared (Bhatt et al., 2022). Appearance, grain type, maturity time, morphological features, size, and seed coat color are enough to characterize different varieties of millets produced (ICRISAT, 2007; Sarita & Singh, 2016). Based on the above‐mentioned quality characteristics, several varieties of millets are being produced worldwide, such as finger, barnyard, proso, kodo, foxtail, pearl, sorghum, and little millet.

Among these climate‐resilient crops, finger millet (FM; *Eleusine coracana*) has been acknowledged so far for its health benefits. FM is an annual herbaceous cereal crop species widely grown and cultivated in regions of Africa as well as Asia. FM belongs to the family *Poaceae* and is said to have originated in the Ethiopia region of Africa, thus being widely cultivated in Africa and South Asia under varied agro‐climatic conditions (Ambati & Sucharitha, 2019). It is one of the crops that were domesticated around 5000 BC and is popularly known as “Ragi” or “Mandua” in India (Karuppasamy, 2015). It has a naked caryopsis grain (1.2–1.8 mm in diameter) with a brick‐red‐colored seed coat, which is highly encouraged in the preparation of traditional foods such as unleavened bread (roti), dumplings (*mudde*), and thin porridge (*ambali*) (Devi et al., 2014). Around 10% of the world's total 30 million tons of millet produced is FM. On worldwide production rates of millet varieties, FM ranks fourth in importance after sorghum, pearl millet, and foxtail millet (Antony Ceasar et al., 2018). The grains are rich in calcium, the most crucial macronutrient for growing children, pregnant women, and the elderly for healthy tissue growth, including the development of strong bones and teeth. It is well reported that whole plant seeds contain 0.34% calcium (Ca), which is comparatively high compared with other cereals (Kumar et al., 2016). FM is found to be effective against hypoglycemia, osteoporosis, hypercholesterolemia, malnutrition, degenerative diseases, and premature aging. It has an anti‐ulcerative property because of its high phytochemical composition (phenols and flavonoids), making it potent for greater utilization in the human diet (Gupta et al., 2017). Although FM grain is a gluten‐free grain with a low glycemic index and high nutraceutical advantages, it is still one of the most neglected and underutilized crop species.

The utilization of FM involves its use as a primitive traditional medicine for the treatment of diseases related to the liver, measles, pleurisy, pneumonia, and smallpox (Bachar et al., 2013). The extraction of starch and polyphenolic components from FM grains is making way for pharmaceutical industries to treat and prepare granules for tablet and capsule dosages (Shiihii et al., 2011). Traditionally, grains are either processed by malting or fermentation, and the resultant extract can be extensively used in weaning or geriatric food (Udeh et al., 2017), beverages, and specific therapeutic products (Subba Rao & Muralikrishna, 2004). Food industries are highly involved in preparing baked products, composite flour, weaning foods, beverages (alcoholic and non‐alcoholic), and non‐beverage products from FM grains (Verma & Patel, 2013). Crops like rice and wheat can help with food security, but FM has higher nutritional qualities in contrast to rice and wheat; hence, it has been suggested that FM may improve the nutritional security in emerging nations in Asia and Africa (Antony Ceasar et al., 2018; Puranik et al., 2017). Considering the potential benefits of FMs, it could be worthwhile to have a complete understanding of millet composition and the processing technologies applied to them. Thus, in the present study, the major emphasis has been on illuminating the nutritional and phytochemical composition, pharmaceutical applications, processing, and food utilization of FM for sustainable growth and development.

## 2 ORIGIN, TAXONOMY, AND GROWTH REQUIREMENTS

FM (*Eleusine coracana*), a self‐fertilized allotetraploid, is said to have originated in the Ethiopia region of Africa. As the name suggests, FM refers to the finger‐like shape of panicles. Taxonomically, FM belongs to the family *Poaceae* with five major races: coracana, vulgaris, elongata, plana, and compacta. FM is popularly known as ragi, mandua, nagli, kapai, marua, nachni, African bird's foot, rapoko, hunsa, wimbi, bulo, telebun, koracan, and kurakkan in the East African highlands. It is revealed that Uganda, Ethiopia, and nearby areas are the primary centers of the foundation of FM, which was brought into India approximately 3000 years ago. The Coracana race is significantly adapted to the arid and semi‐arid climatic conditions of African regions and the Ghats of India. It is commonly cultivated and produced in over 25 countries like Uganda, Nepal, India, Sri Lanka, Bangladesh, East China, Tanzania, Kenya, etc. (Udeh et al., 2017). It follows a hierarchical system: kingdom “Plantae,” class “Magnoliopsida,” order “Poales,” and genus “Eleusine.” FM requires shallow loamy soils as the optimum soil type for growth, a pH of 4.5–7.5 with an annual rainfall of 50 to 60 cm (Kumar et al., 2018), and grows well up to 30–150 cm (FAO, 2017). The agronomic advantages of FM species include their ability to thrive in diverse and adverse environmental conditions, being easy to cultivate, and giving higher yields with good storability.

## 3 PRODUCTION STATISTICS

**FIGURE 1:**
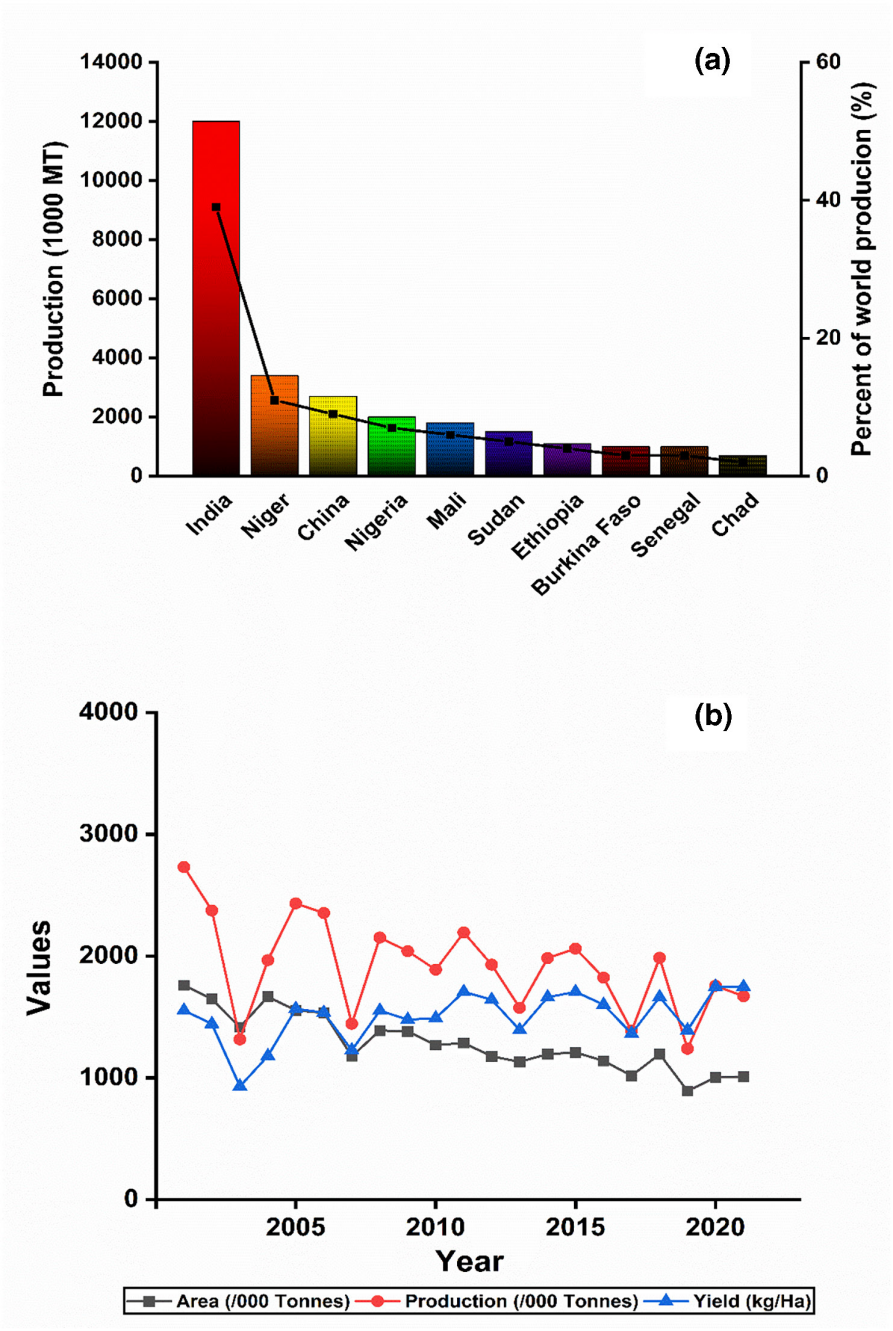
Top‐ten millet‐producing countries regarding their production in 2022 (a) and trend of millet production, area, and yield in India from 1980 to 2022 (b).

**FIGURE 2:**
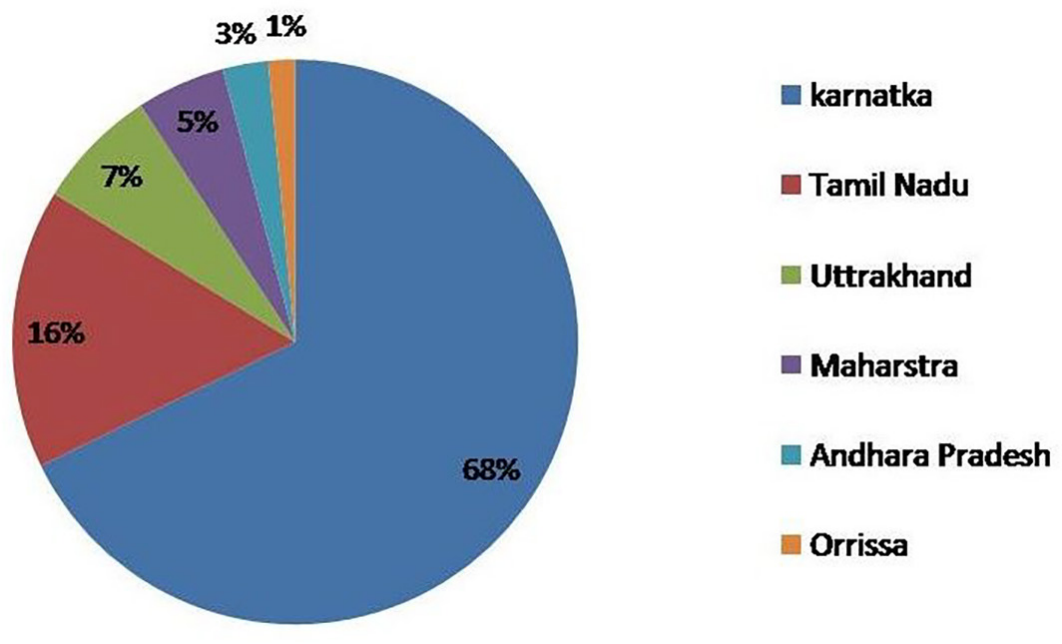
Top‐five finger millet‐producing states in India.

India is the world's largest producer of millet grains, followed by the African countries of Nigeria and Niger. According to FAO, the world production of millets is reported to be 89.17 million metric tons from an area of 74.00 million hectares in 2020 (Rao, 2022). As per the reports, African and Asian countries each provided 43.72% and 52.25% of the world's millet production in 2014 (Kumar, Gattupalli, Babu, & Bhatia, 2020). Figure 1 depicts the top 10 millet‐producing countries regarding their production in 2022 (Index Mundi, 2022) and trends of millet production, area, and yield in India from 1980 to 2020, respectively (Gowri & Shivakumar, 2020). India is the world's largest producer and global leader in production, with a 15% share of the world's total millet (Ambre et al., 2020). India also leads the FM‐producing market, followed by Niger, China, Nigeria, Mali, Sudan, Ethiopia, Senegal, and Chad. About 70%–80% of the world's FM is produced in India and Africa (Ganapathy, 2017). According to the 2017–2018 production data analysis, Karnataka leads the market of FM production in India, accounting for 58% of sharing (Chandra et al., 2016), followed by Tamil Nadu, Uttrakhand, Maharashtra, Andhra Pradesh, Orissa, Jharkhand, and West Bengal. The 2020 production of FM reveals that Karnataka leads the production by producing 1164.06 metric tons, followed by Tamil Nadu (274.50), Uttrakhand (120.12), Maharashtra (87.24), Andhra Pradesh (44.88), and Orrisa (26.24) (IIMR, 2023, millet stats). Figure 2 depicts the production analysis for the year 2020 of the top 5 FM‐producing states in India (IIMR, 2023, millet stats).

## 4 NUTRITIONAL AND PHYTOCHEMICAL COMPOSITION OF FINGER MILLET

### 4.1 Nutritional composition

Nutritionally, FM is considered a good source of essential nutrients. FM is substantially rich in minerals, thus being declared more micronutrient dense than rice and wheat. Table 1 depicts the nutritional composition attributed to FM grain. The millet contains about 65%–75% carbohydrates, 5%–8% protein, 15%–20% dietary fiber, 2.5%–3.5% minerals, and 1%–2% ether extractives (a portion of dry matter extracted with ether) (Devi et al., 2014). The major portion of carbohydrates in FM includes starch (60%), pentosans (6.2%–7.2%), cellulose (1.4%–1.8%), and lignins (0.04%–0.6%) (Hassan et al., 2021). Furthermore, FM also contains a higher amount of total dietary fiber (17.10%–18.90%) and insoluble dietary fiber (13%–15.70%) than wheat and rice. FMs' dietary fibers mainly include non‐starch polysaccharides, non‐α‐glucan oligosaccharides, resistance starches, some polyols, and modified starches (Lafiandra et al., 2014). Non‐starchy polysaccharides are primarily made up of “arabinoxylans” (principal non‐starch compound), with a small amount of β‐d‐glucans, contributing the main component of the fraction of soluble dietary fiber in grain (Udeh et al., 2017). FM is primarily composed of prolamin, whose proportion ranges from 24.6 to 36.2% of the total protein (Lupien, 1990). Although it contains less protein content than other millets, it is enriched with a balanced amino profile and higher sulfur‐containing amino acids. It is estimated that 44.7% of the amino acids in FM are essential amino acids, which is higher than wheat, rice, and the FAO reference protein (33.9%). There are abundant amounts of lysine and methionine in FM, which are lacking in other plant diets (Abioye et al., 2022). In relation to nutritional value, the protein quality score of FMs is 52 higher than that of sorghum (37) and the protein efficiency ratio is 0.95, comparable to that of control casein (1.9) (Shobana et al., 2013). The low fat content in FM (1.3%–1.8%) makes it suitable for a good shelf life. The amounts of free, bound, and structural lipids in FM are 2.2%, 2.4%, and 0.6%, respectively. Regarding fatty acids, they comprise two beneficial polyunsaturated fatty acids, linoleic acid and α‐linolenic acid, which play a vital role in metabolism and the normal development of the central nervous system. The major fatty acids found in decorticated FM, oleic acid (50.43%), palmitic acid (26.18%), and linoleic acid (20.26%), are the most abundant, while stearic acid (0.12%) and linolenic acid (2.60%) are present in relatively low concentrations (Mitharwal et al., 2021).

**TABLE 1:** Nutritional composition of finger millet.

Parameter	Subcategory	Composition	References
Moisture	—	10%–12%	
Protein	Total crude protein Prolamins Globulins and albumins	8%–12% 35%–50% of total crude 8%–15% of total crude	
Fat	Crude fat	1.30%–1.80%.	Devi et al. (2014); Verma and Patel (2013); Chandra et al. (2016); Gull et al. (2016); Singh and Raghuvanshi (2012); Ramashia et al. (2019); Hiremath and Geetha (2019); Ambre et al. (2020); Karki et al. (2020); Hassan et al. (2021)
Carbohydrates	Starch Total carbohydrates Free sugars Cellulose and hemicelluloses Amylose Amylopectin Pentosans Lignin	59% 75%–85% 1%–2% 20%–30% 15%–20% 80%–85% 6.20%–7.20% 0.04%–0.60%	
Ash	Total ash	2.50–3.00 g/100 g	
Energy	Calorific value	300–350 kcal	
Minerals	Calcium (Ca) Phosphorous (P) Iron (Fe) Magnesium (Mg) Sodium (Na) Potassium (K) Copper (Cu) Manganese (Mn) Zinc (Zn)	340–398 mg/100 g 130–250 mg/100 g 3.00–1.90 mg/100 g 137 mg/100 g 11–50 mg/100 g 408 mg/100 g 0.47 mg/100 g 5.49 mg/100 g 2.30 mg/100 g	
Fiber	Dietary fiber Crude fiber Soluble dietary fiber Insoluble dietary fiber Hemi‐cellulose A Hemi‐cellulose B	19.0–20.0 g/100 g 2.60–3.50 g/100 g 9.90 g/100 g 19.70% 1.40% 1.90%	Ofosu et al. (2020); Wang et al. (2022)
Vitamins	Thiamine (B1) Riboflavin (B2) Niacin (B3) Alpha‐tocopherol (E)	0.42 mg/100 g 0.19 mg/100 g 1.10 mg/100 g 22 mg/100 g	
Essential amino acids	Arginine Histidine Lysine Tryptophan Phenylalanine Tyrosine Methionine Cysteine Threonine Leucine Isoleucine Valine	0.30 g/100 g 0.13 g/100 g 0.22 g/100 g 0.10 g/100 g 0.31 g/100 g 0.22 g/100 g 0.21 g/100 g 0.14 g/100 g 0.24 g/100 g 0.69 g/100 g 0.40 g/100 g 0.48 g/100 g	
Fatty acids	Polyunsaturated fatty acids Polysaturated fatty acids Fatty acids Palmitic acid Oleic acid Linoleic acid Linolenic acid	74.40% 25.60% g/100 g of total lipids 21.10–24.70 g/100 g 49.80 g/100 g 24.20 g/100 g 1.30–4.40 g/100 g	
Anti‐nutritional compounds	Tannins (brown variety)	0.10%–2.30%	
Phenolic compounds	Total phenols Major bound phenolic (ferulic acid) Major free phenolic acid (proto‐catechuic acid) TPC TFC Tannins	102 mg/100 g 18.60 mg/100 g 45.0 mg/100 g 136.40 ± 7.07 mg FAE/100 g 115.80 ± 9.1 mg CEQ/100 g 17.65 ± 3.95 mg CEQ/100 g	

It is also the richest source of calcium (344 mg/100 g of FM) and potassium (408 mg/100 g FM) among all other cereal crops like brown rice, wheat, or maize (Gull et al., 2014). It is also reported to contain an appropriate amount of iron (3.9 mg/100 g). Both water‐soluble and fat‐soluble vitamins, vitamin B1 (thiamin), vitamin B2 (riboflavin), vitamin B3 (niacin), and vitamin E (α‐tocopherols), are present in significant amounts (Kumar et al., 2016). It was reported that FM levels of vitamin B1, B2, and B3 were 0.38 mg/100 g, 0.14 mg/100 g, and 1.1 mg/100 g, respectively, which are equivalent to 35%, 12%, and 8% of the recommended daily allowance for healthy adults (Taylor & Kruger, 2016).

### 4.2 Phytochemical composition

The word “phytochemical” refers to a wide variety of physiologically or biologically active natural substances with beneficial medicinal health effects. Phytochemicals are generally characterized as chemicals produced by plants that are essential in terms of health‐promoting factors. Several pieces of evidence support the idea that diets rich in fruit, vegetables, legumes, whole grains, and nuts are beneficial only due to the higher amounts of phytochemicals they contain (Panche et al., 2016).

FM is an important minor cereal that is becoming increasingly popular as a functional food ingredient due to its polyphenol content and dietary fiber content. The nutrients and bioactive components of FM are among the highest of all cereals, compared with the main cereals like rice and wheat. Studies have shown that FM exhibits a diversified variety of phenolic compounds, but phenolic acids and flavonoids are found to be more proportionally and widely studied for their effective extraction and utilization. FM comprises a higher proportion of phenolic compounds in the coat of the seed (0.8%) than in the flour (6.2%) (Chandrasekara & Shahidi, 2011). The phenolic acids and tannins represent the major polyphenols, while flavonoids are found in smaller amounts. Vanillic acid (20.0 μg/g), gallic acid (3.91–30.0 μg/g), syringic (10.0–60.0 μg/g), salicylic (5.12–413.0 μg/g), protocatechuic acid (119.8–405.0 μg/g), p‐hydroxybenzoic (6.3–370.0 μg/g), caffeic (5.9–10.4 μg/g), sinapic (11.0–24.8 μg/g), p‐coumaric acid (1.81–41.1 μg/g), trans‐cinnamic acid (35–100.0 μg/g), and ferulic acid (41–405.0 μg/g) in this proportion are well recognized for their health benefits (Hithamani & Srinivasan, 2014; Udeh et al., 2017). Table 1 depicts the phytochemical composition attributed to FM. FMs contain a high level of tannin, which serves as a barrier to fungus infestation and allows the grains to survive fungal infections. A variety of flavonoids are identified in the seed coat of FM in their soluble form in an amount of 2100 μg/g, which is comparatively higher than other millets (Chandrasekara & Shahidi, 2011). The major flavonoids reported in millet are quercetin, catechin, gallocatechin, epicatechin, epigallocatechin, proanthocyanidins, or condensed tannins (Udeh et al., 2017). According to Xiang et al. (2019), catechin and epicatechin dominated the free fraction of FM, while ferulic acid and trans‐p‐coumaric acid dominated the bound fraction.

The polyphenol content of FM varies considerably depending on the variety. The study conducted by Ramachandra et al. (1977) investigated 32 varieties of FM originating from both India and Africa, containing brown and white seed coats. The polyphenol content of brown varieties ranges from 1.2% to 2.3%, whereas white varieties contain 0.3% to 0.5%. In Indian varieties, the content of polyphenols ranged from 0.08% to 0.96%, whereas it was 0.54% to 3.4% in African varieties. In another study, two types of FMs (Ravana & Osadha) were evaluated for their total phenolic content, which varied from 0.39 to 1.05 mg CE/g (Lansakara et al., 2020). FM may also contain phytates, polyphenols, tannins, trypsin inhibitors, and some dietary fiber, which were earlier termed as “anti‐nutrients” or “nutritional inhibitors” due to their metal chelating and enzyme inhibition properties (Devi et al., 2014), but can be further removed by several processing technologies for effective utilization as health foods or nutraceuticals.

## 5 PHARMACOLOGICAL SIGNIFICANCE

Due to the high amount of bioactive chemicals (phenolic compounds, such as ferulic acid, caffeic acid, vanillic acid, gallic acid, and quercetin) present in the seed coat of millets, several studies have confirmed their significance and role as antioxidant, anti‐carcinogenic, anti‐inflammatory, antiviral, and neuroprotective against fatal disorders such as cancer, cardiovascular disease, diabetes, high blood pressure, cholesterol, and neurodegenerative diseases (Rao et al., 2018). FM phenolics are well reported to work against the inhibition of collagen glycation and cross‐linking, nephritis, diabetes, wound healing, oxidation, enzyme inhibition, osteoporosis, and microbial action. Table 2 depicts the in vitro and in vivo studies conducted on the pharmacological importance of FM.

**TABLE 2:** Pharmacological studies of finger millets and their potential action mechanisms.

S. No.	Property	Test model involved	Dose/concentration/method	Potential findings	References
1	Inhibition of collagen glycation and cross‐linking property	In vitro rat tail tendons (RTT's) test model	3 mg methanolic extract of finger millet for 10 days	Tendons were first incubated with 50 mM glucose and then fed with finger millet extract at ambient temperature as well as pH. Early glycation by pepsin inhibited collagen glycation with 89% solubility and 0.47 dL/g intrinsic viscosity, which may be primarily due to natural antioxidants (phenolic nature) from seed coats.	Hegde et al. (2002); Hegde and Chandra (2005); Devi et al. (2014)
2	Anti‐lithiatic effect (Nephroprotective)	In vivo male albino rats test model	300 mg/kg body weight with Alcoholic extracts of *Eleusine coracana* grains.	Aqueous and alcoholic extracts of finger millet confirmed the reduction in elevated levels of urinary oxalate, showing a potential regulatory action on endogenous oxalate synthesis.	SK and Sudha (2012)
				Also resulted in hyperoxaluria (high oxalate in urine) along with increased renal excretion of calcium and phosphates that led to the deposition of stone‐forming constituents in the kidneys of calculogenic rats.	Shobana et al. (2009) Bahuguna et al. (2009)
3	Anti‐diabetic (Low Glycemic response)	In vivo Streptozotocin‐induced diabetic rat model	20% Finger millet seed coat matter with diet for 6 weeks	Partial inhibition of amylase and α‐glucosidase in enzymatic hydrolysis of polysaccharides ultimately delays the glucose absorption in the body, hence controlling the absorption of glucose.	Rajasekaran et al. (2004)
				Reduced amount of fasting blood glucose levels (hyperglycemia).	
				Phenolic compounds in the seed coat of finger millet may regulate glucose uptake from the intestinal lumen by inhibiting complex carbohydrate digestion as well as absorption, thus maintaining normal blood glucose levels.	Devi et al. (2014)
4	Wound healing and antioxidant property	In vivo Hyperglycemic rat test model	50 g/100 g finger millet (FM), 2 weeks before feeding	The effects of a millet‐based diet were seen on full‐thickness excision skin wounds, and thus it was concluded that the healing process was accelerated by an increased rate of wound contraction in hyperglycemic rats fed rather than non‐diabetic control rats. Antioxidant levels of skin glutathione (GSH), ascorbic acid, and α‐tocopherol were also reported to be lower in alloxan‐induced rats when compared with non‐diabetic rats.	Rajasekaran et al. (2004) Devi et al. (2014)
		Excision wound rat model	300 mg aqueous paste once (16 days)	Significant increase in protein and collagen and decrease in lipid peroxides. A 90% rate of wound contraction was observed, compared with 75% for untreated control rats. After 13 days, a complete closure of wounds was seen, whereas it took 16 days for non‐treated rats.	Pore and Magar (1976)
5	Anti‐osteoporosis	8 children	156 g whole finger millet‐based diet	The effect of the incorporation of a finger millet‐based diet in children on calcium retention ability was studied. The whole finger millet‐based diet has more retention (26%), which is 224 mg/854 mg of the diet, than the refined finger millet (25%) which is 175 mg/692 mg of the diet.	Kurien and Doraiswamy (1967)
6	Anti‐microbial property	Fungal genera (in vitro studies)	Acidic methanol extract of finger millet seed coat	Resistance of finger millet grain towards fungal attack, which is attributed to tannins present in the seed coat of the grain. It turned out to be physical barriers to the invasion by fungus (not specified). The acidic methanolic extracts from the seed coat have been reported to show higher antibacterial and antifungal activity as compared to whole flour extract, as phenolics (tannins) reside more in the seed coat than in the whole grain.	Viswanath et al. (2009) Devi et al. (2014)
7	Enzyme inhibitory property (α‐glucosidase and pancreatic amylase)	Rat intestinal acetone powder (in vitro studies)	α‐glucosidase from rat intestine (100 mg)	Seed coat phenolics showed strong inhibition towards α‐glucosidase and pancreatic amylase, with IC_50_ values of 16.9 and 23.5 μg of phenolics for α‐glucosidase and pancreatic amylase, respectively.	Shobana et al. (2009)
		Enzyme kinetic studies		FM seed coat polyphenols inhibited (non‐competitive mode) aldose reductase extracted from cataracted human eye lenses. Quercetin from finger millet was found to be more effective in inhibition with an IC_50_ of 14.8 nM.	Shobana et al. (2013)
8	Cholesterol‐lowering	Alloxan‐induced diabetic rat model	55% FM incorporated in the daily diet (28 days)	Study depicted a 13% reduction in serum cholesterol levels when compared to the control rat model.	Hegde and Chandra (2005)
		Male albino rats fed	FM diet for 8 weeks	Significant lower serum cholesterol (65 mg/dL) was observed as compared to normal diet‐fed animals (95 mg/dL).	Shobana et al. (2013)

## 6 FINGER MILLET PROCESSING

**FIGURE 3:**
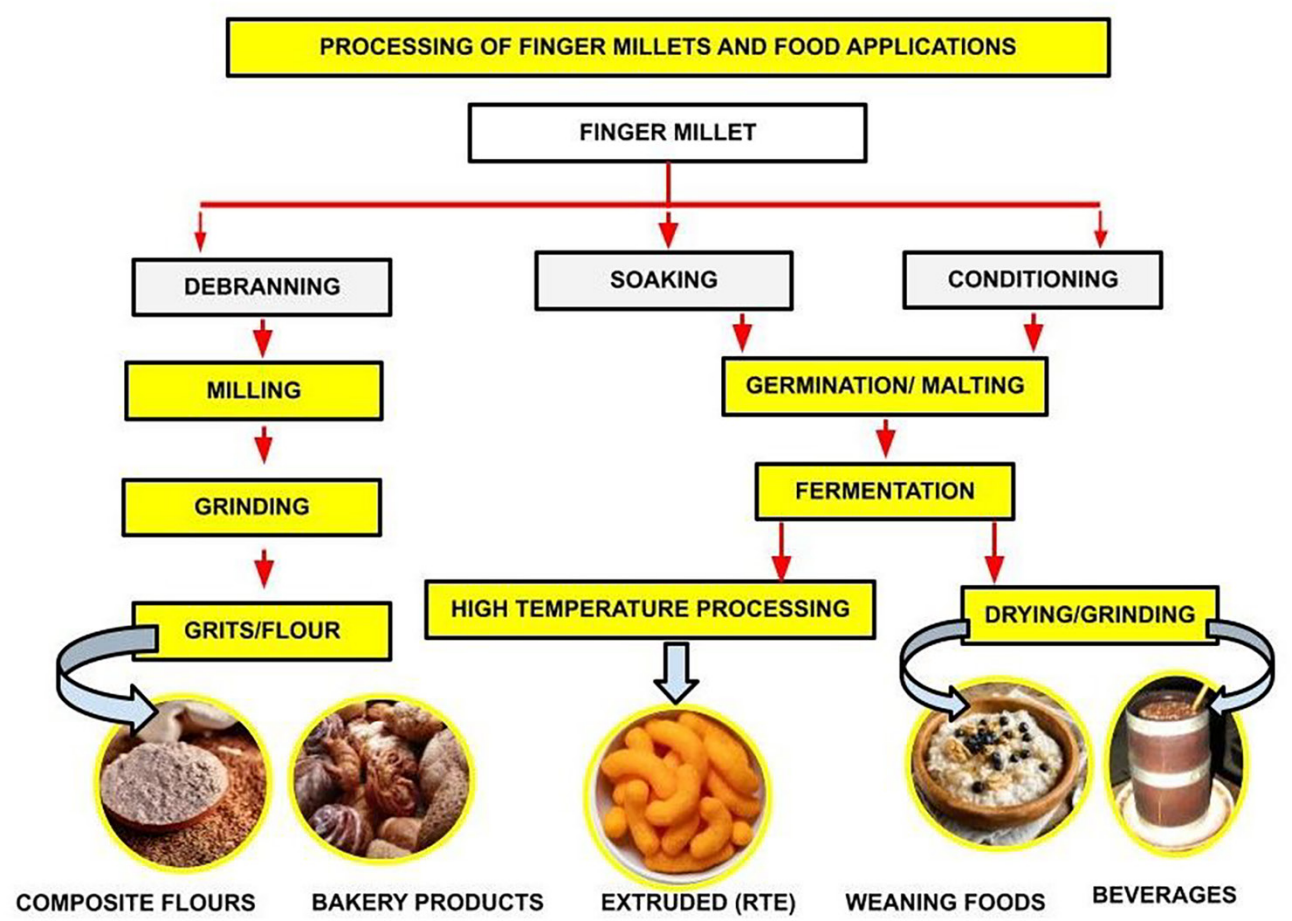
Processing methods of finger millets and their food applications.

Processing is defined as the conversion of raw materials into finished edible products after several treatments through advanced and traditional processing means. Food processing methods are developed and highly encouraged as they make the final product more appealing and improve its nutritive value (Singh & Raghuvanshi, 2012). Presently, FM is consumed as a staple food by only some of society, but its appealing nutritional characteristics have now created interest among researchers to explore its nutraceutical properties (Kumar et al., 2016). Before going to the processing plant, every grain must undergo several preliminary steps such as cleaning, grading, size reduction, and separation to make them free from unwanted materials (stones, soil particles, stalks, chaffs, and grains of other crops; Ambre et al., 2020). Traditional processing of grains may include soaking/germination, malting, fermentation, milling/grinding, roasting, and popping/puffing, which is practiced in rural areas. Extrusion processing of FM grains is one of the modern‐day techniques emerging for millet processing. Thus, the use of modern‐day processing technology in manufacturing commercial millet‐based products is highly recommended. Figure 3 represents the different processing methods applied to FMs for their value addition in different food applications.

### 6.1 Physical processing treatments

#### 6.1.1 Soaking

Soaking is not considered a processing treatment but can be regarded as a pre‐treatment before the final processing of millet grain. Soaking is an essential step in many treatments, including germination, cooking, extraction, and fermentation. Alternatively, it can also be referred to as steeping. The process results in the leaching of several soluble molecules, such as oligosaccharides, tannins, and phytic acid. Soaking adds distilled water to FM grains until the grains are fully steeped in water and left overnight at an ambient temperature of 30 to 60°C (Saleh et al., 2013). Soaking involves immersing solid grains into hot or lukewarm water and then drying them in a hot air oven at 60° for 90 min with the major objective of quick, uniform water absorption and increasing the bioavailability of zinc (Ramashia et al., 2019). During the soaking process, water is absorbed, which enhances heat transfer during cooking. As a result, various antinutritional factors are effectively inactivated (Wang et al., 2022). Furthermore, grain hydration is essential for microbial growth during fermentation to maintain the required water activity. After soaking raw FM for 36 h at ambient temperature, the trypsin inhibitor activity decreased from 6.37 to 5.70 TUI/mg (Yenasew & Urga, 2022). Hotz and Gibson (2007) have reported that soaking FM grains for 1–2 days at room temperature of about 25°C in 1:10 parts of water has a reducing effect on polyphenols, phytate, saponins, oxalates, and trypsin inhibitors, which ultimately improve the bioavailability and bio‐accessibility of minerals and also the nutrition quality. A study depicted that when soaked in NaOH solution or distilled H_2_O for 8 h, the tannin content in ragi is reduced. Syeunda et al. (2021) also reported a decreased condensed tannin content of 39%–55% in germinated FM due to the leaching of tannins during soaking. The period of soaking also had a significant effect on the decrease in anti‐nutritional compounds. The phytic acid content was reduced from 250 mg/100 g during 12 h of soaking to 221 mg/100 g during 48 h of soaking (Shigihalli et al., 2018). Hithamani and Srinivasan (2014) have reported a significant reduction in total phenolic content (10.2–5.95 mg/g), bioaccessible polyphenol (2.65–1.84 mg/g), total flavonoids (5.54–1.78 mg/g), bio‐accessible flavonoids (1.09–0.89 mg/g), and tannin (5.93–3.57 mg/g), along with increased percentage bioaccessibility of polyphenols (25.5%–30.9%) and flavonoids (5.54–1.78 mg/g), after pressure cooking of FM (15 psi for 15–20 min). Dharmaraj and Malleshi (2011) studied the effect of hydrothermal treatment on FM, thereby improving protein and carbohydrate digestibility, which increased from 61 to 73 g/100 g for carbohydrates and 79 to 91 g/100 g for proteins.

#### 6.1.2 Milling/grinding/decortications

Milling/grinding is one of the important preliminary steps in the processing of millets, which involves size reduction and the removal of coarse bran or seed coat. These millets comprise a large portion of husk/bran, which needs to be removed by dehusking and debranning before consumption (Singh & Raghuvanshi, 2012). The milling process involves sorting, cleaning, hulling, debranning, and kilning for further processing (Rasane et al., 2015). FM kernels possess a fragile endosperm and an intact seed coat, which makes them difficult to polish and cook in the form of grain or grit like rice or other cereals. Therefore, flour must always be prepared by pulverizing or milling the grain. 10% of water is usually added during the milling process to facilitate the removal of the hard, fibrous husk. Milling improves the removal of some antinutrients (phytates and tannins), thereby improving the bioavailability of iron. After milling, a significant reduction of polyphenols by 74.7% and phytate phosphorus by 39.8% was observed in FM (Ramashia et al., 2019).

In the case of FM, the debranching and decortication methods previously used for most cereals were not effective due to the intact seed coat and highly fragile endosperm. Consequently, the soft endosperm of FM is hardened through hydrothermal processing (hydration, steaming, and drying) so that it will be able to withstand the mechanical forces during decortication. Decorticated FM can be prepared as discrete grains, similar to rice. The seed coat matter produced as a by‐product during decortation contains numerous health‐promoting compounds. Several food formulations can benefit from the inclusion of seed coat matter as a source of fiber and micronutrients (Shobana et al., 2013).

### 6.2 Biochemical processing treatments

#### 6.2.1 Germination

Germination is a traditional process initiated to soften the kernel structure by soaking for 2–24 h and then spreading on a damp cloth for up to 24–48 h or incubating at 30°C for 48 h to ease the processing and increase the nutritional composition of millet grains (Shimray et al., 2012). To enhance the nutritional composition of the seed and enhance the functional properties of the seed, moisture, oxygen, and a favorable temperature are necessary for germination. Compared with other methods, it retains most minerals and some vitamins (Abioye et al., 2022). It is a biochemical enrichment tool that involves the seed's transition from dormant to active state (Ghavidel & Prakash, 2007). A study depicted that germination increased the germination percentage, loss, and shoot length of FM grain, thereby improving the nutritional content of the flour obtained (increased protein content, fiber content, total titratable acidity, color, pH, fat, and decreased carbohydrate content). A reduction in total carbohydrate content and an increase in the activity of amylase are the primary functions of germination, produced by the breakdown of carbohydrates in the first phase of germination (Oghbaei & Prakash, 2016; Zhang et al., 2015). Mbithi‐Mwikya et al. (2002) also reported a decrease in starch content upon germination. The major difference is seen in the protein content of germinated FMs, which increased rapidly due to the production of new enzymes and the synthesis of amino acids during germination. Moreover, the enhanced enzymatic activity leads to higher protein digestibility and a reduction of anti‐nutritional content, thereby improving the bioavailability of various essential minerals (Nguyen et al., 2022). It has also been reported that germination decreases pH and increases the total titrable acidity of FMs. pH and total titrable acidity were affected by catabolic reactions of complex organic compounds (lipids and proteins), while the acidity increased because of hydrolysis (Owheruo et al., 2019). The hydrolytic enzymes produced during germination result in the hydrolysis of complex substances such as phytate, polypeptides, and lipids, which produce acid phosphate, amino acids, and fatty acids. Consequently, products made from germinated FM are more digestible (Nefale & Mashau, 2018). Apart from enriching the nutritional value, germination also helps lower FM's anti‐nutritional content, as tannins, phytic acid, and trypsin inhibitor activity, thus improving the bioavailability of minerals (Rathore et al., 2019; Yenasew & Urga, 2022). The study also depicts that the phytic acid content in FM decreased from 676.77 mg/100 g to 587.20 mg/100 g during 12 h of soaking and later reduced to 238.46 mg/100 g after 36 h of germination at 37°C (Patel & Dutta, 2018). In another study, 48 h germination led to a 13%–44% reduction of phytic acid in five varieties of FMs, ranging from 787.05–1283.92 mg/100 g to 577.20–838.69 mg/100 g. The decreased phytic acid content might be due to the degradation of phytate to inorganic phosphorus and inositol by the phytase enzyme. Additionally, condensed tannin content also decreased by 39%–55% in germinated FMs due to the leaching of tannins and enhanced activity of polyphenol oxidase (Syeunda et al., 2021). Oxalate is an important anti‐nutritional compound that hinders the absorption of calcium from FMs leached out during the steeping period of germination and leads to decreased oxalic acid content (Suma & Asna, 2017). Prolonged germination may result in a reduction of fat and high malt loss. Chauhan (2018) have concluded that a 24‐h germination period is well suited to retaining maximum profit.

Hithamani and Srinivasan (2014) reported a 33.5% decrease in the total phenolic content of FMs upon germination from 10.2 ± 0.21 to 6.71 ± 1.08 mg/g. Similarly, a 34%–53% decrease in TPC was also observed in five varieties of FMs when subjected to 48 h germination (Syeunda et al., 2021). In another study, a decrease in total phenols (42.63%–38.46%) and tannins (61.66%–33.33%) was reported, whereas antioxidant activity and total flavonoid content were increased by 26.66%–33.33% and 48.30–51.13%, respectively. The antioxidant activity of FMs germinated for 96 h increased from 70%–72.14% (DPPH assay) to 46.91–53.54 mg/g (FRAP assay). A major cause of higher antioxidant activity is the release of bound phenolics by enzymatic action, structural changes, and depolymerization of high molecular weight phenolic compounds at the time of germination. The alkaloids of FM were also increased by germination (36.03%–68.44%) (Antony Ceasar et al., 2018).

#### 6.2.2 Fermentation

Fermentation refers to a metabolic process involving chemical changes through the action of enzymes. Food production and process technology involve converting complex carbohydrates into simpler sugars in the presence of certain enzymes, with/without oxygen. The activity of microorganisms brings about a desirable change in either foodstuffs or any beverages. FM can also be processed by fermentation. Traditionally, FM is consumed as porridges, pancakes, and beverages, which involve fermentation as a major processing step (Singh & Raghuvanshi, 2012). Fermentation of FM using different cultures has been studied, while lactic acid fermentation has been reported to promote biological value (BV), net protein utilization (NPU), and certain vitamins (thiamin, riboflavin, and niacin), which can be further utilized for developing health foods (infant food, weaning food, beverages, and confectionary items) (Malleshi, 2007). Basappa et al. (1997) reported a significant increase in the concentration of the vitamin profile in fermented FM: niacin—4.2 mg/100 g, pantothenic acid—1.6 mg/100 g, and riboflavin—0.62 mg/100 g. *Lactobacillus salivaricus*, an essential yeast in the fermentation of FM, increases the lysine and tryptophan content by 7.1% and 17.8%, respectively (Rathore et al., 2019).

### 6.3 Thermal/hydrothermal processing treatments

#### 6.3.1 Popping/roasting/puffing

Popping/puffing/roasting is one of the processing treatments that aid in the conversion of raw grain seeds into fine flour. Roasting hardens the kernel and reduces the water content, which easily releases the endosperm for flour, thereby increasing the shelf life. Popping or puffing involves the high heat treatment (70–80°) given to grains and the bursting of endosperm out of the grain in a non‐specific or deformed shape. Popping/puffing has also been reported to increase in vitro nitrogen and starch digestion. The digestibility of proteins is influenced by heating, making proteins more susceptible to hydrolysis. High enzymatic digestion is achieved by increased starch digestibility due to the degree of starch gelatinization and the release of starch granules from the protein matrix (Singh & Raghuvanshi, 2012). The study depicted that popping has a significant effect on iron content; it significantly increases the availability of nutrients. Another study depicted that during the roasting of FM, functional and phytochemical compositions such as moisture, fat, protein, phenols, and antioxidant activity tend to decrease slightly, whereas total carbohydrate content, ash, and fiber increase along with the increase in the bioavailability of minerals like calcium and iron (Singh et al., 2018). Popped/roasted FM products have been reported to improve the pleasant aroma, increasing the acceptable taste and quality of grains by inactivating destructive bacteria (Thapliyal & Singh, 2015). Wadikar et al. (2007) concluded in a study that the change in fatty acid composition was non‐significant when different FM varieties were conditioned for 2 h with 20% moisture content and puffed at 220–230°C. The neutral lipids decreased by 9.3%, with an increase in glycolipids by 21.92% and phospholipids by 33.3% (Jaybhaye et al., 2014). Auko (2009) has revealed that FM, when subjected to roasting at different temperatures and time combinations and converted to flour/porridge, decreased in viscosity by 50%–60% in roasted FM.

#### 6.3.2 Parboiling

Parboiling is the hydrothermal treatment given to cereal/millets before decortications to achieve maximum recovery of grains without nutrient losses. It is carried out in 3 major steps: soaking, steaming, and drying (Rathore et al., 2019). Soaking is done at an ambient temperature of about 70°C within a time period of 10 to 24 h, ensuring the complete absorption of water. It is reported that soaking whole grains for a longer period can decrease phytic acid content by 20% to 30% (Sene et al., 2018). Soaking is followed by a steaming process, which gelatinizes the starch in the endosperm of the millet grains at elevated temperatures in either autoclaves or steamers. Finally, an open‐sun or tray drying technique is carried out to reduce the final moisture (10% acceptable). Parboiling ensures the easy extraction of endosperm from the bran, and many nutrients rich in the seed coat also penetrate the seed through soaking. Varadharaju and Ganesan (2017) have reported that parboiling treatment has an effect on reduction in the glycemic index and increases residual starch content, hence making it a suitable pre‐treatment technique to make millets as prebiotic ingredients (Kaushik et al., 2021).

#### 6.3.3 Extrusion

Extrusion is an HTST (high‐temperature short‐time) thermal shear process used to transform raw material into a processed finished product where food is cooked by employing high pressure, temperature, and rotating screws when forcefully passed through a die (Nikmaram et al., 2017). Extrusion cooking is widely applied in the production of snack foods, which helps retain significant nutrients and provides high textural properties to the product. Biochemical transformations usually take place during extrusion cooking, such as starch gelatinization, denaturation of proteins, increased mineral bioavailability, and destruction of anti‐nutritional factors. The final extruded product is affected by several factors, such as the feed's moisture content, barrel temperature, and screw speed (Kaur & Prasad, 2021). Rathore et al. (2019) have reported that FM flour (16%–18% M.C.) can extrude in the barrel (at a temperature of 100–120°), exhibiting an excellent expansion index in terms of porosity, crunchiness, and smoother texture. The effects of different processing treatments on FM are discussed in Table 3.

**TABLE 3:** Effects of processing treatments on finger millet grains.

Processing treatment	Effects associated	References
Milling/grinding	Facilitates the easy removal of fibrous husk, thereby improving the bioavailability of iron and removing phytates and tannins. Converts the grain into flour, separates the bran, germs, and endosperm. Reduces the microbial population on the grains.	Ertop et al. (2020)
Soaking	Increases the quick and uniform water absorption capability of the grains and also improves the bioavailability of zinc. Trypsin activity gets inhibited. Reduction of trypsin inhibitor activity (TIA), oxalates, polyphenols, and saponins by 33%; tannins were reduced by 20%; and phytates by 39.47 to 24.17% at 25°C for 12 h.	Ramashia et al. (2019); Rathore et al. (2019); Yenasew and Urga (2022)
Germination	Softens the kernel's structure, thereby easing the processing. Lowers the anti‐nutritional content, thus improving the bioavailability of minerals. A significant reduction in amylase activity, trypsin inhibitors, phytates by 54%, and tannins by 65%. Increase in nutrient composition was significant at 25°C for 48 h.	Rathore et al. (2019)
Fermentation	Promotes the biological value (BV), net protein utilization (NPU) and increases certain vitamins. Improves taste and nutritional properties. Reduction of phytates, tannins, and trypsin inhibitors by 20%, 52%, and 32%, respectively.	Singh and Raghuvanshi (2012)
Popping/roasting/puffing	Hardens the kernel and reduces the water content, facilitating the easy release of the endosperm for flour purposes, thereby increasing the shelf life. Popping increases the availability of iron, calcium, and other nutrients; reduction in antioxidant activity also occurs. Reduction of tannin content by 54% and phytates by 41%. In vitro nitrogen and starch digestibility was also increased at 25°C and 19% moisture content.	Singh et al. (2018)
Parboiling	Maximum grain recovery is achieved with less nutrient loss. Decreases phytic acid content and improves the easy extraction of endosperm from bran. Reduces the glycemic index and increases residual starch content.	Sene et al. (2018); Kaushik et al. (2021)
Extrusion	Exhibits excellent expansion properties such as porosity, crunchiness, and smoother texture. Increases mineral bioavailability and reduces nutritional content. Finger millet grain can be processed easily for the preparation of precooked and dehydrated foods.	Rathore et al. (2019); Kaur and Prasad (2021)

## 7 UTILIZATION OF FINGER MILLET FOR TRADITIONAL AND NOVEL FOODS

Despite its high nutritional content and climate‐resilient properties, FM still lacks processing and production. One of the major reasons has always been the unavailability of specific processing technologies and machinery to produce RTC and RTE products on the market. But now, R&D sectors are paying more attention towards processing and value addition of millets for nutritional benefits and enhancing shelf life. FM can be used as a whole or as a substitution with other cereal grains (wheat, rice, maize, etc.). Some of the commonly used processing technologies, such as milling, malting, baking, popping, fermenting, roasting, and a combination of these treatments (Karki et al., 2020) can produce several value‐added products, which in turn will enhance the consumption of FM and make it appealing as well as valuable. Table 4 depicts the traditional and modern‐day applications of FM in food systems.

**TABLE 4:** Modern and traditional applications of finger millet in food systems.

Application	Product	Composition	Potential findings	Sources
Modern applications				
Baked products	Composite flour biscuits	Finger millet:wheat flour (70:30 and 60:40)	Results showed 60:40 (w/w) compositions retained a good quality of biscuits. Breaking strength and expansion ratio of the biscuit were higher at 70:30 (w/w).	Saha et al. (2011)
	Composite flour muffins (FM)	Finger millet: wheat flour (60:40) Malted finger millet: wheat flour (20:80)	The blend of additives with plain and malted finger millet flour has considerably good texture and sensory characteristics. Muffins are rich in protein (9.12%–10.34%) and mineral content (163.22–164.36 mg/100 g [Ca] and 3.46–6.32 mg/100 g [Fe])	Ladkat et al. (2019)
	Cakes	Different ratios of wheat and finger millet flour (0%–25%)	Wheat and finger millet flour (80:20) had the best sensory characteristics.	Taynath et al. (2018)
Extruded products	Noodles	30%–50% FM flour blended with wheat flour	Finger millet‐based noodles have beneficial advantages for diabetic patients due to their low glycemic response (45.13) as compared to control (62.59). 30% FM‐incorporated noodles have good nutritional value with efficient noodle formation.	Shukla and Srivastava (2014)
	Extruded Snack	FM, corn flour, rice flour (40:30:30), and banana powder with different combinations	Desirable process parameters were 119°C barrel temperature, 346 rpm screw speed, and 3.67 g banana powder. Composite flour has a good expansion ratio, water‐holding capacity, and higher overall acceptability compared to control.	Sukumar and Athmaselvi (2019)
	Pasta	20% FM flour blended with composite flour (pearl millet, semolina, and carrot pomace).	Finger millet‐based pasta improved dietary fiber, minerals, and antioxidant profiles. It also improved the cooking weight, swelling index, and water absorption of pasta.	Gull et al. (2016)
	Noodles	Rice flour and FM flour (0%–100%) in different ratio	Rice flour: FM flour (80:20) had the highest sensory scores. Blended flour had high nutritional characteristics and enhanced antioxidant activity.	Chen et al. (2021)
Weaning food	Weaning food Premix	Soaked rice, germinated and roasted green gram, finger millet (25:37.5:37.5)	High protein (16.05%) and mineral content. Good sensory qualities with a shelf life of 6 months. Premix can be used as a cheap source of essential nutrients for children, pregnant women, and lactating women.	Jadhavar et al. (2022)
	Porridge	Termite powder: finger millet (18.6:81.4)	Significant increase in protein content from 8 to 11.5%. No toxic heavy metals were detected. Developed porridge to combat nutritional deficiencies.	Musundire et al. (2021)
	Weaning mix	Composite flour with 4%, 6% and 8% finger millet flour	Rich in protein, fiber, and minerals compared with a wheat‐based market mix. 6% finger millet addition has more acceptability.	Prasanna et al. (2020)
Beverages	Functional beverage	Milk‐based. Enriched with oats and finger millets (50%–90%)	Finger millet: oats (60:40) was best in terms of sensory analysis. Rich in dietary fiber, β‐glucan, and mineral content.	Kumar, Kaur, Tomer, Rasane, and Gupta (2020)
	RTD beverage	Sorghum and finger millet in different proportions with other flavor ingredients	High in total phenolic content and antioxidant activity, with rich nutritional properties. All variants are above 7 in 9‐point hedonic scale.	Bembem and Agrahar‐Murugkar (2020)
	Symbiotic beverage	Germinated finger millets: oats: potable water (15:10:75) 4 flour variants were prepared (plain, functional milk, rose, and marigold)	Rose‐flavored fermented with *L.acidophilus* has the most desirable characteristics, with a probiotic count of 6.38 logCFU/mL. A low cholesterol content was detected. Good antioxidant activity with high sensory acceptability	Kumar, Kaur, and Tomer (2020)
Traditional applications				
Fermented products	Madua		Golden yellow in color, sweet, and has a good alcoholic flavor prevalent in Arunachal Pradesh. Fermented with starter culture, the perforated basket was covered with Ekam leaves for 4–7 days.	Zvauya et al. (1997); Ilango and Antony (2014); Kubo (2016); Kumar et al. (2018); Hassan et al. (2021) Rotela et al. (2021)
	Togwa		Combination of maize meal and finger millet malt is prevalent in Tanzania and Nigeria.	
	Kunun zaki		Another fermented traditional beverage belongs to Zimbabwe and is made of milk and finger millet malt, which produces a highly nutritious product.	
	Sur		FM‐based fermented beverage prepared in the Lug valley of Kullu, Kangra, and Barot valleys, of district Mandi, Himachal Pradesh, India. A mixture (inocula) of roasted barley and local herbs known as ‘dhaeli’ is used to carry out fermentation. The millet is kneaded with water to make dough and left in a container for 7–8 days for natural fermentation. The product has been reported to have 5%–10% alcohol	
	Chang		Semi‐alcoholic popular Tibetan fermented FM drink in the Ladakh region of India	
	Koozh		Fermented drink made out of pearl or finger millet flour and rice, eaten by ethnic groups in Tamil Nadu.	
	Kodo ko jaanr		A traditional mild‐alcoholic beverage prepared from FM, consumed in Eastern Himalayan areas of the Darjeeling hills and Sikkim, India.	
	Koozh		Fermented beverages made with FM flour and rice are consumed mainly by ethnic communities in Tamil Nadu, India. Prepared by two fermentation stages.	
	Themsing		Prepared by blending finger millet, bong/barley, or a mixture of both.	
	Rakshi		Prepared from rice, barley, finger millet, and maize. Grains are cooked, spread on ‘charang’, or bamboo (involves fermentation for 2–3 days), and then extracted by distillation.	
	Mingri and lohpani		Alcoholic fermented beverage of Arunachal Pradesh, prepared from finger millet/barley. The first liquid obtained after 4–5 days of fermentation is called beer from mingri, and the remaining filtrate again undergoes fermentation; a darker yellow liquid is obtained known as lohpani.	
	Ragi malt		Milk‐based prepared drink made from malted FM flour mixed with skim milk powder or whole milk powder, sugar, and flavoring substances. The prepared beverage is reported to be a good source of nutrients and thus can be utilized as energy/health drinks.	
	Masvusvu		Sweet beverage traditionally made from malted finger millet, found in Zimbabwe.	
	Mangisi		Sweet–sour product naturally produced by fermentation of sieved masvusvu.	
	Pombe		Fermented alcoholic drinks in Tanzania using finger millets, namely kimpumu, komoni, and kiambule germinated finger millet, have a strong taste.	

### 7.1 Fermented products

**FIGURE 4:**
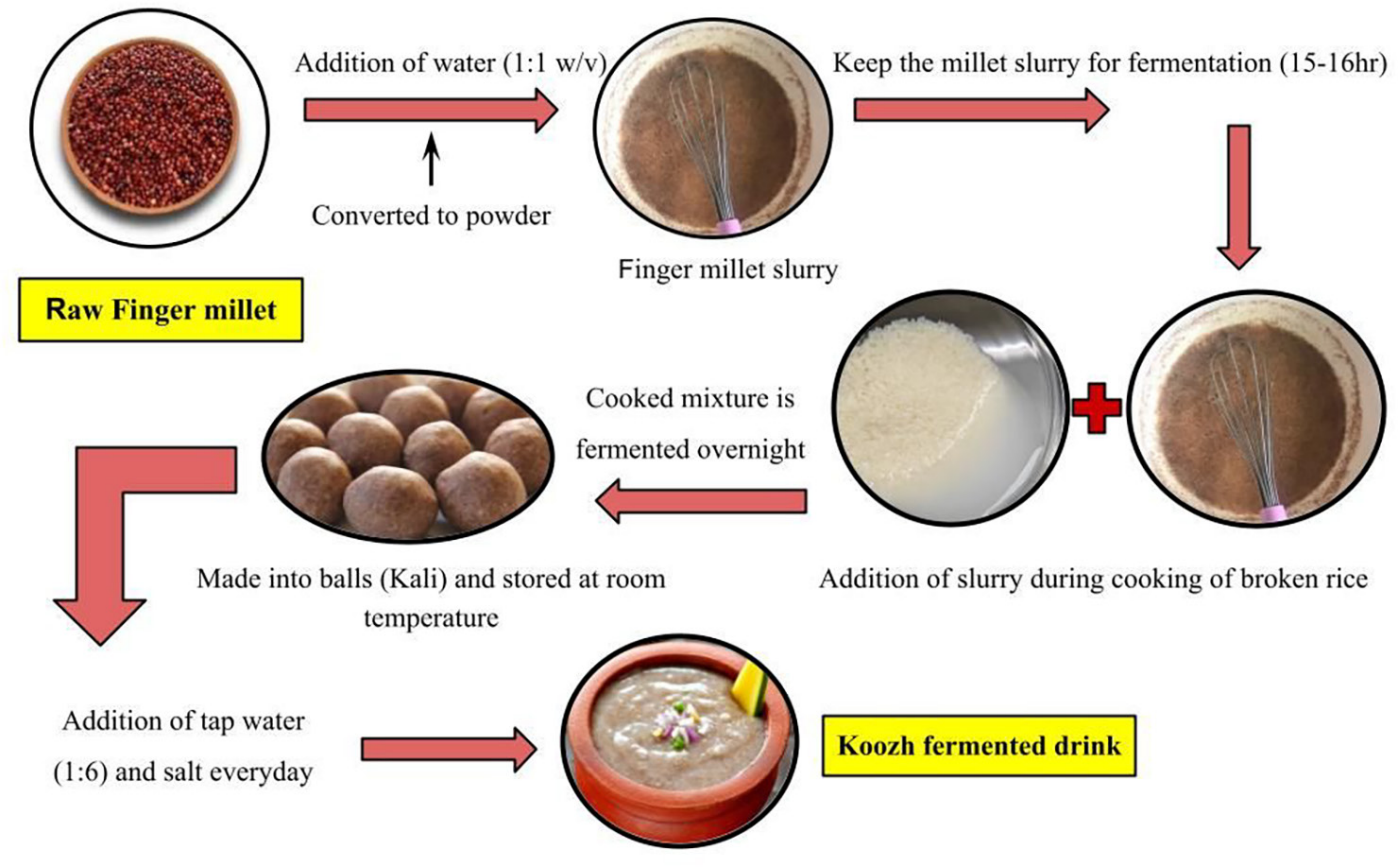
Flow diagram for the preparation of Koozh.

Fermented foods made with FM are highly accepted as they improve the taste and enrich the food with fiber, calcium, and protein content, significantly reducing anti‐nutritional compounds. Fermented FM‐based foods can also be prepared using either malted or sprouted grains, which enhance taste and flavor. Traditionally, FM was highly encouraged to be used to formulate fermented soft drinks and beer. Madua is a famous traditional fermented drink that hails from Arunachal Pradesh. It has a golden yellow color and sweet, good alcoholic flavor that uses Ekam leaves for its fermentation (Rotela et al., 2021). Togwa is also one of the same kinds formulated from the combination of maize meal and FM malt prevalent in Tanzania and Nigeria. Kunun zaki, a highly nutritious traditional beverage in Zimbabwe, is made of milk and FM malt (Ilango & Antony, 2014). “Sur” is an FM‐based fermented beverage prepared in the Lug valley of Kullu, Kangra, and Barot valleys of Mandi, Himachal Pradesh, India. It uses barley and other herbs for fermentation (Kumar et al., 2018). “Koozh” is another fermented beverage made with FM flour and rice with two fermentation stages (Figure 4). It is highly consumed by ethnic communities in Tamil Nadu, India (Shrivastava et al., 2012). New and recent advancements have led to the formulation of composite flours for bread, dough, biscuits, cookies, and many more. Mythrayee and Pavithra (2017) have concluded in their research that enhancement of nutritive content was observed in FM‐based composite bread fermented with baker's yeast and LAB (lactic acid bacteria). Bread fermented with baker's yeast had a higher moisture content (21.24%) and vitamin B content (1.09 mg), along with a longer shelf life expectancy. Another study reveals the formulation of FM and skimmed milk‐based powder gruel. A thick‐consistency product was obtained when composite powders of FM (0%–100%) were inoculated by bacterial cultures at 45°C and stored at 7°C.

### 7.2 Weaning foods

Weaning foods are regarded as a supplement to breast milk, which is made to provide the correct proportion of proteins, energy, and nutrition for the growth and development of children or infants. Several weaning foods (e.g. Cerelac and Ceregrow) are being introduced in the market with the same purpose of supporting early growth and development in children and are made out of cereals, fruit pulps, and flour. The major methods used to develop weaning food formulations are extrusion, roasting, fermentation, and sprouting. FM malt was traditionally utilized for infant feeding due to its good malting characteristics and exceptional nutritional properties (Palatability). FM malt is used as a cereal base for low‐dietary bulk and calorie‐dense weaning foods, supplementary foods, health foods, and amylase‐rich foods. Verma and Patel (2013) studied the formulation of a healthy mix from FM flour soaked in water at room temperature of 25°C for 48 h. A drying operation was carried out, and the temperature was maintained below 75°C with 10%–12% moisture content. Malted grains were roasted at 70–80°C to develop the product's characteristic aroma and desired quality. The obtained malt was pulverized and passed through a sieve, which was then mixed with skim milk powder or whole milk powder, sugar, and flavors. The prepared milk‐based powder/gruel is called “ragi malt,” which is ultimately a good source of nutrients. Several other studies have successfully formulated weaning mixes out of varied flour varieties such as sorghum, pearl millet, and FM flours. Malleshi (2007) formulated a weaning mix using 60% of each above‐mentioned flour, 30% of mung bean flour, and 10% of non‐fat dry milk. A similar weaning mix was formulated using kodo, foxtail, finger, and little millets in varied ratios, resulting in a mix rich in protein, ash, calcium, and crude fiber compared with wheat‐based market samples without fortification.

### 7.3 Extruded foods

A healthy market for noodles has emerged in India and other countries due to the changing eating patterns of children and teenagers. The popularity of FM noodles, particularly, is rising as people become more aware of their nutritious benefits (Ambre et al., 2020). Extrusion in food processing is a technique that consists of forcing a mixture of soft, well‐mixed ingredients through an opening or die designed to develop a specific product. Pasta, noodles, and spaghetti are some extruded food items, also known as convenience foods, prepared through a cold extrusion system (hard after drying). Several studies and trials are being conducted on the preparation of noodles from different combinations of FM, like FM and wheat (1:1); finger millet with wheat and soy flour in the ratio of 5:4:1 resulted in better quality noodles with high nutritional value as well (Gull et al., 2014). It is not easy to replace wheat when preparing noodles, as the presence of wheat gluten helps in easy extrusion and gives a smooth and fissure‐free texture. Another study reveals the formulation of FM‐based pasta (20% with wheat flour), which improved pasta's dietary fiber, minerals, and antioxidant profile (Verma & Patel, 2013).

### 7.4 Bakery products

Bakery goods such as biscuits, nankhatai, muffins, cakes, and breads can also be prepared by incorporating FM flours, and many attempts and efforts have been made to standardize the recipe and product quality. Millets are superior to other cereal grains in terms of fibers and micronutrients that can be used in bakery industries. In a recent study, many attempts have been made to improve the nutritional quality of cakes with minerals and fiber content using malted FM flour (Desai et al., 2010). Saha et al. (2011) formulated biscuits from composite flours with different proportions of a FM:wheat flour (70:30 and 60:40). Biscuits were analyzed based on dough characteristics. Results revealed that a composite flour of 60:40 FM:wheat is better for quality. The bread was also prepared from composite flours (e.g., FM with wheat, proso millet, and barnyard millet). Sudha et al. (1998) formulated muffins by replacing wheat flour with FM flour (0%–100%). A blend of additives with 60% FM flour improved the muffin's volume and quality attributes.

## 8 CONCLUSION AND FUTURE PROSPECTS

As far as the nutritional health benefits of millets are concerned, millets are regarded as the richest sources of antioxidants, calcium, proteins, dietary fibers, and carbohydrates. Scientists have successfully conducted numerous studies (in vitro and in vivo) that have proven to be beneficial to human growth. The millet sector, usually in India, has faced numerous challenges limited to production, processing, value addition, marketing, and consumption, which have hindered the overall consumption and popularity of millet worldwide. Though FAO has declared 2023 as the “International Year of Millets” to subsequently increase the production and productivity of millets throughout the globe, other staple crops such as rice, wheat, and maize have high carbon emission rates as compared to millets. Therefore, the value addition of FM should be encouraged to formulate maximum nutrition‐rich products. The lack of gluten (less strength) in millets has limited their usage in bakery products, but it can be substituted with some other cereal crops to gain an advantage. Further research and development are required to produce value‐added/functional foods, thus making FM a promising crop for sustainable world development. The biotechnology and food processing industries must play a vital role in value addition and new product development for maximum profits.

## AUTHOR CONTRIBUTIONS

**Rhythm Kalsi:** Conceptualization (equal); data curation (equal); investigation (equal); validation (equal); visualization (equal); writing – original draft (equal). **Jasleen Bhasin:** Conceptualization (equal); data curation (equal); investigation (equal); software (equal); validation (equal); visualization (equal); writing – original draft (equal). **Gulden Goksen:** Conceptualization (equal); formal analysis (equal); software (equal); visualization (equal); writing – original draft (equal); writing – review and editing (equal). **Piyush Kashyap:** Conceptualization (equal); data curation (equal); investigation (equal); supervision (equal); visualization (equal); writing – review and editing (equal).

## ACKNOWLEDGEMENTS

Not applicable.

## FUNDING INFORMATION

No funding was provided for this review paper.

## CONFLICT OF INTEREST STATEMENT

The authors declare that there are no conflicts of interest. The data that support the findings of this study are available from the corresponding author upon reasonable request.

## Data Availability

The data that support the findings of this study are available from the corresponding author upon reasonable request.

## References

[fsn33659-bib-0001] Abioye, V. F. , Babarinde, G. O. , Ogunlakin, G. O. , Adejuyitan, J. A. , Olatunde, S. J. , & Abioye, A. O. (2022). Varietal and processing influence on nutritional and phytochemical properties of finger millet: A review. Heliyon, 8, e12310. 36590554 10.1016/j.heliyon.2022.e12310 PMC9800331

[fsn33659-bib-0002] Ambati, K. , & Sucharitha, K. V. (2019). Millets‐review on nutritional profiles and health benefits. International Journal of Recent Scientific Research, 10(7), 33943–33948.

[fsn33659-bib-0111] Ambre, P. K. , Sawant, A. A. , & Sawant, P. S. (2020). Processing and value addition: A finger millet review. Journal of Pharmacognosy and Phytochemistry, 9(2), 375–380.

[fsn33659-bib-0003] Antony Ceasar, S. , Maharajan, T. , Ajeesh Krishna, T. P. , Ramakrishnan, M. , Victor Roch, G. , Satish, L. , & Ignacimuthu, S. (2018). Finger millet [*Eleusine coracana* (L.) Gaertn.] improvement: Current status and future interventions of whole genome sequence. Frontiers in Plant Science, 9, 1054. 30083176 10.3389/fpls.2018.01054 PMC6064933

[fsn33659-bib-0004] Auko, J. C. (2009). Effects of roasting on the nutritional quality of finger millet. Unpublished Masters' Thesis. Makerereb University.

[fsn33659-bib-0110] Bachar, K. , Imangour, E. , Khaled, A. B. , Abid, M. , Haddad, M. , Yahya, L. B. , El Jarray, N. , & Ferchichi, A. (2013). Fibre content and mineral composition of the finger millet of the Oasis of Gabes of Tunisia. Journal of Agricultural Science, 5(2), 219–226.

[fsn33659-bib-0005] Bahuguna, Y. M. , Rawat, M. S. M. , Juyal, V. , & Gnanarajan, G. (2009). Antilithiatic effect of grains of *Eleusine coracana* . Saudi Pharmaceutical Journal, 17(2), 182–188.

[fsn33659-bib-0006] Basappa, S. C. , Somashekar, D. , Agrawal, R. , Suma, K. , & Bharathi, K. (1997). Nutritional composition of fermented ragi (chhang) by phab and defined starter cultures as compared to unfermented ragi (*Eleucine coracana* G.). International Journal of Food Sciences and Nutrition, 48(5), 313–319.

[fsn33659-bib-0007] Bembem, K. , & Agrahar‐Murugkar, D. (2020). Development of millet‐based ready‐to‐drink beverage for geriatric population. Journal of Food Science and Technology, 57(9), 3278–3283. 32728276 10.1007/s13197-020-04359-9 PMC7374645

[fsn33659-bib-0008] Bhatt, D. , Rasane, P. , Singh, J. , Kaur, S. , Fairos, M. , Kaur, J. , Gunjal, M. , Mahato, D. K. , Mehta, C. , Avinashe, H. , & Sharma, N. (2022). Nutritional advantages of barnyard millet and opportunities for its processing as value‐added foods. Journal of Food Science and Technology, 1–13. 10.1007/s13197-022-05602-1 PMC10497464 37711577

[fsn33659-bib-0009] Bommy, D. , & Maheswari, S. K. (2016). Promotion of millets cultivation through consumption. International Journal of Current Research Academic Review, 3, 74–80.

[fsn33659-bib-0010] Chandra, D. , Chandra, S. , & Sharma, A. K. (2016). Review of finger millet (*Eleusine coracana* (L.) Gaertn): A power house of health benefiting nutrients. Food Science and Human Wellness, 5(3), 149–155.

[fsn33659-bib-0011] Chandrasekara, A. , & Shahidi, F. (2011). Determination of antioxidant activity in free and hydrolyzed fractions of millet grains and characterization of their phenolic profiles by HPLC‐DAD‐ESI‐MSn. Journal of Functional Foods, 3(3), 144–158.

[fsn33659-bib-0112] Chauhan, E. S. (2018). Effects of processing (germination and popping) on the nutritional and anti‐nutritional properties of finger millet (*Eleusine coracana*). Current Research in Nutrition and Food Science Journal, 6(2), 566–572.

[fsn33659-bib-0012] Chen, J. , Wang, L. , Xiao, P. , Li, C. , Zhou, H. , & Liu, D. (2021). Informative title: Incorporation of finger millet affects in vitro starch digestion, nutritional, antioxidative and sensory properties of rice noodles. LWT, 151, 112145.

[fsn33659-bib-0013] Desai, A. D. , Kulkarni, S. S. , Sahoo, A. K. , Ranveer, R. C. , & Dandge, P. B. (2010). Effect of supplementation of malted ragi flour on the nutritional and sensorial quality characteristics of cake. Advance Journal of Food Science and Technology, 2(1), 67–71.

[fsn33659-bib-0014] Devi, P. B. , Vijayabharathi, R. , Sathyabama, S. , Malleshi, N. G. , & Priyadarisini, V. B. (2014). Health benefits of finger millet (*Eleusine coracana* L.) polyphenols and dietary fiber: A review. Journal of Food Science and Technology, 51, 1021–1040. 24876635 10.1007/s13197-011-0584-9 PMC4033754

[fsn33659-bib-0015] Dharmaraj, U. , & Malleshi, N. G. (2011). Changes in carbohydrates, proteins and lipids of finger millet after hydrothermal processing. LWT‐Food Science and Technology, 44(7), 1636–1642.

[fsn33659-bib-0017] Ertop, M. H. , Bektaş, M. , & Atasoy, R. (2020). Effect of cereals milling on the contents of phytic acid and digestibility of minerals and protein. Ukrainian Food Journal, 9(1), 136–147.

[fsn33659-bib-0018] FAO (Food and Agriculture Organization) . (2017). World food situation. Retrieved January 25, 2023, from http://www.fao.org/worldfoodsituation/csdb/en/

[fsn33659-bib-0019] Gaikwad, V. , Rasane, P. , Singh, J. , Idate, A. , & Kumthekar, S. (2021). Millets: Nutritional potential and utilization. Pharma Innovation, 10(5), 310–313.

[fsn33659-bib-0020] Ganapathy, K. N. (2017). Improvement in finger millet: Status and future prospects. In V. Patil (Ed.), Millets and sorghum: Biology and genetic improvement (pp. 87–111). John Wiley & Sons.

[fsn33659-bib-0021] Ghavidel, R. A. , & Prakash, J. (2007). The impact of germination and dehulling on nutrients, antinutrients, in vitro iron and calcium bioavailability and in vitro starch and protein digestibility of some legume seeds. LWT‐Food Science and Technology, 40(7), 1292–1299.

[fsn33659-bib-0022] Gowri, M. U. , & Shivakumar, K. M. (2020). Millet scenario in India. Economic Affairs, 65(3), 363–370.

[fsn33659-bib-0023] Gull, A. , Ahmad, N. G. , Prasad, K. , & Kumar, P. (2016). Technological, processing and nutritional approach of finger millet (*Eleusine coracana*)—A mini review. Journal of Food Processing & Technology, 7(593), 2.

[fsn33659-bib-0024] Gull, A. , Jan, R. , Nayik, G. A. , Prasad, K. , & Kumar, P. (2014). Significance of finger millet in nutrition, health and value added products: A review. Journal of Environmental Science, Computer Science and Engineering & Technology, 3(3), 1601–1608.

[fsn33659-bib-0025] Gupta, S. M. , Arora, S. , Mirza, N. , Pande, A. , Lata, C. , Puranik, S. , Kumar, J. , & Kumar, A. (2017). Finger millet: A “certain” crop for an “uncertain” future and a solution to food insecurity and hidden hunger under stressful environments. Frontiers in Plant Science, 8, 643. 28487720 10.3389/fpls.2017.00643 PMC5404511

[fsn33659-bib-0026] Hassan, Z. M. , Sebola, N. A. , & Mabelebele, M. (2021). The nutritional use of millet grain for food and feed: A review. Agriculture & Food Security, 10, 1–14. 10.1186/s40066-020-00282-6 PMC8005370 33815778

[fsn33659-bib-0027] Hegde, P. S. , & Chandra, T. S. (2005). ESR spectroscopic study reveals higher free radical quenching potential in kodo millet (*Paspalum scrobiculatum*) compared to other millets. Food Chemistry, 92(1), 177–182.

[fsn33659-bib-0028] Hegde, P. S. , Chandrakasan, G. , & Chandra, T. S. (2002). Inhibition of collagen glycation and crosslinking in vitro by methanolic extracts of finger millet (*Eleusine coracana*) and Kodo millet (*Paspalum scrobiculatum*). The Journal of Nutritional Biochemistry, 13(9), 517–521. 12231421 10.1016/s0955-2863(02)00171-7

[fsn33659-bib-0029] Himanshu, K. , Sonawane, S. K. , & Arya, S. S. (2018). Nutritional and nutraceutical properties of millets: A review. Clinical Journal of Nutrition and Diet, 1(1), 1–10.

[fsn33659-bib-0030] Hiremath, S. P. , & Geetha, K. (2019). Nutritional composition of raw, malted and popped finger millet (*Eleusinecoracana*) varieties. International Journal of Current Microbiology and Applied Sciences, 8(2), 966–974.

[fsn33659-bib-0031] Hithamani, G. , & Srinivasan, K. (2014). Effect of domestic processing on the polyphenol content and bioaccessibility in finger millet (*Eleusine coracana*) and pearl millet (*Pennisetum glaucum*). Food Chemistry, 164, 55–62. 24996305 10.1016/j.foodchem.2014.04.107

[fsn33659-bib-0032] Hotz, C. , & Gibson, R. S. (2007). Traditional food‐processing and preparation practices to enhance the bioavailability of micronutrients in plant‐based diets. The Journal of Nutrition, 137(4), 1097–1100. 17374686 10.1093/jn/137.4.1097

[fsn33659-bib-0033] ICRISAT . (2007). International Crops Research Institute for the Semi‐Arid Tropics, 2007 annual report. http://test1.icrisat.org/Publications/EBooksOnlinePublications/Annul‐report2007.pdf

[fsn33659-bib-0034] IIMR . (2023). Millet stats. Retrieved January 12, 2023, from https://www.milletstats.com/finger‐millet‐ragi/

[fsn33659-bib-0035] Ilango, S. , & Antony, U. (2014). Assessment of the microbiological quality of koozh, a fermented millet beverage. African Journal of Microbiology Research, 8(3), 308–312.

[fsn33659-bib-0036] Index mundi (2022). Retrieved January 27, 2023, from https://www.indexmundi.com/agriculture/?commodity=millet&graph=production

[fsn33659-bib-0037] Jadhavar, R. S. , Jaiswal, S. G. , & Bornare, D. T. (2022). Development of weaning food premixes for infants based on Ragi, green gram and Rice. International Journal of Food and Nutritional Sciences, 11, 96–104.

[fsn33659-bib-0038] Jaybhaye, R. V. , Pardeshi, I. L. , Vengaiah, P. C. , & Srivastav, P. P. (2014). Processing and technology for millet based food products: A review. Journal of Ready to Eat Food, 1(2), 32–48.

[fsn33659-bib-0040] Karki, A. , Chandra, A. , Joshi, S. , Rawat, P. , & Sharma, S. (2020). An overview of finger millet (*Eleusine coracana* L.). Journal of Pharmacognosy and Phytochemistry, 9(4), 866–869.

[fsn33659-bib-0041] Karuppasamy, P. (2015). Overview on millets. Trends in Biosciences, 8(13), 3269–3273.

[fsn33659-bib-0042] Kaur, R. , & Prasad, K. (2021). Technological, processing and nutritional aspects of chickpea (*Cicer arietinum*)—A review. Trends in Food Science & Technology, 109, 448–463.

[fsn33659-bib-0043] Kaushik, N. , Yadav, P. , Khandal, R. K. , & Aggarwal, M. (2021). Review of ways to enhance the nutritional properties of millets for their value‐addition. Journal of Food Processing and Preservation, 45(6), e15550.

[fsn33659-bib-0044] Kubo, R. (2016). The reason for the preferential use of finger millet (*Eleusine coracana*) in eastern African brewing. Journal of the Institute of Brewing, 122(1), 175–180.

[fsn33659-bib-0045] Kumar, A. , Kaur, A. , & Tomer, V. (2020). Process optimization for the development of a synbiotic beverage based on lactic acid fermentation of nutricereals and milk‐based beverage. LWT, 131, 109774.

[fsn33659-bib-0046] Kumar, A. , Kaur, A. , Tomer, V. , Rasane, P. , & Gupta, K. (2020). Development of nutricereals and milk‐based beverage: Process optimization and validation of improved nutritional properties. Journal of Food Process Engineering, 43(1), e13025.

[fsn33659-bib-0047] Kumar, A. , Metwal, M. , Kaur, S. , Gupta, A. K. , Puranik, S. , Singh, S. , Singh, M. , Gupta, S. , Babu, B. K. , Sood, S. , & Yadav, R. (2016). Nutraceutical value of finger millet [*Eleusine coracana* (L.) Gaertn.], and their improvement using omics approaches. Frontiers in Plant Science, 7, 934. 27446162 10.3389/fpls.2016.00934 PMC4925701

[fsn33659-bib-0048] Kumar, A. , Tomer, V. , Kaur, A. , Kumar, V. , & Gupta, K. (2018). Millets: A solution to agrarian and nutritional challenges. Agriculture & Food Security, 7(1), 1–15.

[fsn33659-bib-0049] Kumar, H. M. V. , Gattupalli, N. , Babu, S. C. , & Bhatia, A. (2020). Climate‐smart small millets (CSSM): A way to ensure sustainable nutritional security. In V. Venkatramanan , S. Shah , & R. Prasad (Eds.), Global climate change: Resilient and smart agriculture (pp. 137–154). Springer.

[fsn33659-bib-0050] Kurien, P. P. , & Doraiswamy, T. R. (1967). Nutritive value of refined ragi (*Eleusine coracana*) flour. 2. Effect of replacing cereal in a poor diet with whole or refined ragi flour on the nutritional status and metabolism of nitrogen, calcium and phosphorus in children (boys). Indian Journal of Nutrition and Dietetics, 4, 102–109.

[fsn33659-bib-0051] Ladkat, K. S. , Kotecha, P. M. , Chavan, U. D. , & Bhise, A. H. (2019). Physicochemical properties of finger millet and malted finger millet muffins. BIOINFOLET‐A Quarterly Journal of Life Sciences, 16(1and2), 31–34.

[fsn33659-bib-0052] Lafiandra, D. , Riccardi, G. , & Shewry, P. R. (2014). Improving cereal grain carbohydrates for diet and health. Journal of Cereal Science, 59(3), 312–326. 24966450 10.1016/j.jcs.2014.01.001 PMC4064937

[fsn33659-bib-0053] Lansakara, P. , Liyanage, R. , Jayawardana, B. , & Vidanarachchi, J. (2020). A comparative study on nutritional and nutraceutical properties of finger millet (*Eleusine coracana*) and rice (*Oryza sativa*). World Journal of Biology Pharmacy and Health Sciences, 1(1), 17–24.

[fsn33659-bib-0054] Lupien, J. R. (1990). Sorghum and millets in human nutrition. FAO. ICRISAT. ao.org. 86

[fsn33659-bib-0055] Malleshi, N. G. (2007). Nutritional and technological features of ragi (finger millet) and processing for value addition. Food Uses of Small Millets and Avenues for Further Processing and Value Addition, 9–19.

[fsn33659-bib-0056] Mbithi‐Mwikya, S. , Van Camp, J. , Mamiro, P. R. , Ooghe, W. , Kolsteren, P. , & Huyghebaert, A. (2002). Evaluation of the nutritional characteristics of a finger millet based complementary food. Journal of Agricultural and Food Chemistry, 50(10), 3030–3036. 11982437 10.1021/jf011008a

[fsn33659-bib-0057] Mitharwal, S. , Kumar, S. , & Chauhan, K. (2021). Nutritional, polyphenolic composition and in vitro digestibility of finger millet (*Eleusine coracana* L.) with its potential food applications: A review. *Food* . Bioscience, 44, 101382.

[fsn33659-bib-0058] Musundire, R. , Dhlakama, R. B. , & Serere, J. H. (2021). Physico‐chemical and sensory quality evaluation of an extruded nutrient‐dense termite (*Macrotermes natalensis*) and millet (*Eleusine coracana*) instant porridge. International Journal of Tropical Insect Science, 41, 2059–2070.

[fsn33659-bib-0059] Mythrayee, R. , & Pavithra, A. (2017). Comparative study on nutritive content of finger millet‐wheat composite bread fermented with lactic acid bacilli and yeast. IOSR Journal of Biotechnology and Biochemistry, 3(3), 15–21.

[fsn33659-bib-0060] Nefale, F. E. , & Mashau, M. E. (2018). Effect of germination period on the physicochemical, functional and sensory properties of finger millet flour and porridge. Asian Journal of Applied Sciences, 6(5), 360–367.

[fsn33659-bib-0061] Nguyen, T. H. , Vu, D. C. , Ho, T. H. , Nguyet, N. T. , Tuan, N. N. , Thang, T. D. , Trinh, N. T. N. , & Rose, D. J. (2022). Changes in enzymatic activity and in vitro protein digestibility of four millet varieties upon germination and quality evaluation of cookies prepared from germinated millet composite flours. Journal of Food Processing and Preservation, 46(10), e16854.

[fsn33659-bib-0062] Nikmaram, N. , Leong, S. Y. , Koubaa, M. , Zhu, Z. , Barba, F. J. , Greiner, R. , Oey, I. , & Roohinejad, S. (2017). Effect of extrusion on the anti‐nutritional factors of food products: An overview. Food Control, 79, 62–73.

[fsn33659-bib-0063] Ofosu, F. K. , Elahi, F. , Daliri, E. B. M. , Chelliah, R. , Ham, H. J. , Kim, J. H. , & Oh, D. H. (2020). Phenolic profile, antioxidant, and antidiabetic potential exerted by millet grain varieties. Antioxidants, 9(3), 254. 32245008 10.3390/antiox9030254 PMC7139927

[fsn33659-bib-0064] Oghbaei, M. , & Prakash, J. (2016). Effect of primary processing of cereals and legumes on its nutritional quality: A comprehensive review. Cogent Food & Agriculture, 2(1), 1136015.

[fsn33659-bib-0065] Owheruo, J. O. , Ifesan, B. O. , & Kolawole, A. O. (2019). Physicochemical properties of malted finger millet (*Eleusine coracana*) and pearl millet (*Pennisetum glaucum*). Food Science & Nutrition, 7(2), 476–482. 30847125 10.1002/fsn3.816 PMC6392857

[fsn33659-bib-0066] Panche, A. N. , Diwan, A. D. , & Chandra, S. R. (2016). Flavonoids: An overview. Journal of Nutritional Science, 5, 1–12. 10.1017/jns.2016.41 PMC5465813 28620474

[fsn33659-bib-0067] Patel, S. , & Dutta, S. (2018). Effect of soaking and germination on anti‐nutritional factors of garden cress, wheat and finger millet. International Journal of Pure and Applied Bioscience, 6(5), 1076–1081.

[fsn33659-bib-0068] Pore, M. S. , & Magar, N. G. (1976). Effect of ragi feeding on serum cholesterol level. Indian Journal of Medical Research, 64(6), 909–914. 977045

[fsn33659-bib-0069] Prasanna, M. S. , Sowjanya, V. S. , Jaya, E. , & Rajender, G. (2020). Development of millet based instant weaning mix. Journal of Pharmacognosy and Phytochemistry, 9(4), 1908–1913.

[fsn33659-bib-0070] Puranik, S. , Kam, J. , Sahu, P. P. , Yadav, R. , Srivastava, R. K. , Ojulong, H. , & Yadav, R. (2017). Harnessing finger millet to combat calcium deficiency in humans: Challenges and prospects. Frontiers in Plant Science, 8, 1311. 28798761 10.3389/fpls.2017.01311 PMC5526919

[fsn33659-bib-0071] Rajasekaran, N. S. , Nithya, M. , Rose, C. , & Chandra, T. S. (2004). The effect of finger millet feeding on the early responses during the process of wound healing in diabetic rats. Biochimica et Biophysica Acta (BBA)‐Molecular Basis of Disease, 1689(3), 190–201. 15276645 10.1016/j.bbadis.2004.03.004

[fsn33659-bib-0072] Ramachandra, G. , Virupaksha, T. K. , & Shadaksharaswamy, M. (1977). Relation between tannin levels and in vitro protein digestibility in finger millet (*Eleusine coracana* Gaertn.). Journal of Agricultural and Food Chemistry, 25(5), 1101–1104. 893835 10.1021/jf60213a046

[fsn33659-bib-0073] Ramashia, S. E. , Anyasi, T. A. , Gwata, E. T. , Meddows‐Taylor, S. , & Jideani, A. I. O. (2019). Processing, nutritional composition and health benefits of finger millet in sub‐saharan Africa. Food Science and Technology, 39, 253–266.

[fsn33659-bib-0074] Rao, D. (2022). MILLETS “The Future Super Food for India” The Associated Chambers of Commerce and Industry of India. pp. 16.

[fsn33659-bib-0075] Rao, D. B. , Ananthan, R. , Hariprasanna, K. , Bhatt, V. , Rajeswari, K. , Sharma, S. , & Tonapi, V. A. (2018). Nutritional and health benefits of Nutri cereals (p. 86). ICAR_Indian Institute of Millets Research (IIMR).

[fsn33659-bib-0076] Rasane, P. , Jha, A. , Sabikhi, L. , Kumar, A. , & Unnikrishnan, V. S. (2015). Nutritional advantages of oats and opportunities for its processing as value added foods‐a review. Journal of Food Science and Technology, 52, 662–675. 25694675 10.1007/s13197-013-1072-1 PMC4325078

[fsn33659-bib-0077] Rathore, T. , Singh, R. , Kamble, D. B. , Upadhyay, A. , & Thangalakshmi, S. (2019). Review on finger millet: Processing and value addition. The Pharma Innovation Journal, 8(4), 283–291.

[fsn33659-bib-0078] Rotela, S. , Borkar, S. , & Borah, A. (2021). Health benefits of millets and their significance as functional food: A review. The Pharma Innoation Journal, 10, 158–162.

[fsn33659-bib-0079] Saha, S. , Gupta, A. , Singh, S. R. K. , Bharti, N. , Singh, K. P. , Mahajan, V. , & Gupta, H. S. (2011). Compositional and varietal influence of finger millet flour on rheological properties of dough and quality of biscuit. LWT‐Food Science and Technology, 44(3), 616–621.

[fsn33659-bib-0080] Saleh, A. S. , Zhang, Q. , Chen, J. , & Shen, Q. (2013). Millet grains: Nutritional quality, processing, and potential health benefits. Comprehensive Reviews in Food Science and Food Safety, 12(3), 281–295.

[fsn33659-bib-0081] Sarita, E. S. , & Singh, E. (2016). Potential of millets: Nutrients composition and health benefits. Journal of Scientific and Innovative Research, 5(2), 46–50.

[fsn33659-bib-0082] Sene, S. , Gueye, M. T. , Sarr, F. , Sow, M. S. , Diallo, Y. , & Gaye, M. L. (2018). Optimization of parboiling process pearl millet (*Pennisetum Glaucum* [L.]r.Br.) GB 87‐35 variety. Journal of Food Process Technology, 9(8), 1–4.

[fsn33659-bib-0083] Shigihalli, S. , Ravindra, U. , & Ravishankar, P. (2018). Effect of processing methods on phytic acid content in selected white finger millet varieties. International Journal of Current Microbiology and Applied Sciences, 7(2), 1829–1835.

[fsn33659-bib-0084] Shiihii, S. U. , Musa, H. , Bhati, P. G. , & Martins, E. (2011). Evaluation of physicochemical properties of *Eleusine coracana* starch. Nigerian Journal of Pharmaceutical Sciences, 10(1), 91–102.

[fsn33659-bib-0085] Shimray, C. A. , Gupta, S. , & Venkateswara Rao, G. (2012). Effect of native and germinated finger millet flour on rheological and sensory characteristics of biscuits. International Journal of Food Science & Technology, 47(11), 2413–2420.

[fsn33659-bib-0086] Shobana, S. , Krishnaswamy, K. , Sudha, V. , Malleshi, N. G. , Anjana, R. M. , Palaniappan, L. , & Mohan, V. (2013). Finger millet (Ragi, *Eleusine coracana* L.): A review of its nutritional properties, processing, and plausible health benefits. Advances in Food and Nutrition Research, 69, 1–39. 23522794 10.1016/B978-0-12-410540-9.00001-6

[fsn33659-bib-0087] Shobana, S. , Sreerama, Y. N. , & Malleshi, N. G. (2009). Composition and enzyme inhibitory properties of finger millet (*Eleusine coracana* L.) seed coat phenolics: Mode of inhibition of α‐glucosidase and pancreatic amylase. Food Chemistry, 115(4), 1268–1273.

[fsn33659-bib-0088] Shrivastava, K. , Greeshma, A. G. , & Shrivastava, B. (2012). Biotechnology in action—A process technology of alcoholic beverages is practices by different tribes of Arunachal Pradesh, north East India. Indian Journal of Traditional Knowledge, 11, 81–89.

[fsn33659-bib-0089] Shukla, K. , & Srivastava, S. (2014). Evaluation of finger millet incorporated noodles for nutritive value and glycemic index. Journal of Food Science and Technology, 51, 527–534. 24587528 10.1007/s13197-011-0530-x PMC3931870

[fsn33659-bib-0090] Singh, N. , David, J. , Thompkinson, D. K. , Seelam, B. S. , Rajput, H. , & Morya, S. (2018). Effect of roasting on functional and phytochemical constituents of finger millet (*Eleusine coracana* L.). The Pharma Innovation Journal, 7(4), 414–418.

[fsn33659-bib-0091] Singh, P. , & Raghuvanshi, R. S. (2012). Finger millet for food and nutritional security. African Journal of Food Science, 6(4), 77–84.

[fsn33659-bib-0092] SK, M. , & Sudha, K. (2012). Functional and phytochemical properties of finger millet (*Eleusine coracana* L.) for health. International Journal of Pharmaceutical, Chemical and Biology Sciences, 2(4), 431–438.

[fsn33659-bib-0093] Subba Rao, M. V. S. S. T. , & Muralikrishna, G. (2004). Structural analysis of arabinoxylans isolated from native and malted finger millet (*Eleusine coracana*, ragi). Carbohydrate Research, 339(14), 2457–2463. 15388362 10.1016/j.carres.2004.07.005

[fsn33659-bib-0094] Sudha, M. L. , Vetrimani, R. , & Rahim, A. (1998). Quality of vermicelli from finger millet (*Eleusine coracana*) and its blend with different milled wheat fractions. Food Research International, 31(2), 99–104.

[fsn33659-bib-0095] Sukumar, A. , & Athmaselvi, K. A. (2019). Optimization of process parameters for the development of finger millet based multigrain extruded snack food fortified with banana powder using RSM. Journal of Food Science and Technology, 56, 705–712. 30906028 10.1007/s13197-018-3527-x PMC6400736

[fsn33659-bib-0096] Suma, F. P. , & Asna, U. (2017). Impact of household processing methods on the nutritional characteristics of pearl millet (*Pennisetum typhoideum*): A review. Journal of Food Processing and Technology, 4(1), 28–32.

[fsn33659-bib-0097] Syeunda, C. O. , Anyango, J. O. , Faraj, A. K. , & Kimurto, P. K. (2021). In vitro protein digestibility of finger millet complementary porridge as affected by compositing precooked cowpea with improved malted finger millet. Journal of Food Science and Technology, 58, 571–580. 33568850 10.1007/s13197-020-04569-1 PMC7847920

[fsn33659-bib-0098] Taylor, J. R. N. , & Kruger, J. (2016). Millets. In B. Caballero , P. M. Finglas , & F. Toldr'a (Eds.), Encyclopedia of food and health (1st ed., pp. 748–757). Academic Press.

[fsn33659-bib-0099] Taynath, S. J. , Adhau, G. W. , & Said, P. P. (2018). Development and sensory evaluation of ragi‐wheat composite cake. Current Research in Nutrition and Food Science Journal, 6(1), 142–147.

[fsn33659-bib-0100] Thapliyal, V. , & Singh, K. (2015). Finger millet: Potential millet for food security and power house of nutrients. International or Research in Agriculture and Forestry, 2(2), 22–33.4.26664951

[fsn33659-bib-0101] Udeh, H. O. , Duodu, K. G. , & Jideani, A. I. O. (2017). Finger millet bioactive compounds, bioaccessibility, and potential health effects—A review. Czech Journal of Food Sciences, 35, 7–17.

[fsn33659-bib-0102] Varadharaju, N. , & Ganesan, S. (2017). Effect of parboiling (thermal treatment) on de‐hulling and cooking qualities of little millet (*Panicum sumatrense*) and foxtail millet (*Setaria italica*). Journal of Nutrition and Food Science, 7(3S), 56.

[fsn33659-bib-0103] Verma, V. , & Patel, S. (2013). Value added products from nutri‐cereals: Finger millet (*Eleusine coracana*). Emirates Journal of Food and Agriculture, 25(3), 169–176.

[fsn33659-bib-0104] Viswanath, V. , Urooj, A. , & Malleshi, N. G. (2009). Evaluation of antioxidant and antimicrobial properties of finger millet polyphenols (*Eleusine coracana*). Food Chemistry, 114(1), 340–346.

[fsn33659-bib-0113] Wadikar, D. D. , Premavalli, K. S. , Satyanarayanaswamy, Y. S. , & Bawa, A. S. (2007). Lipid profile of finger millet (*Eleusine coracana*) varieties. Journal of Food Science and Technology‐Mysore, 44(1), 79–81.

[fsn33659-bib-0105] Wang, H. , Fu, Y. , Zhao, Q. , Hou, D. , Yang, X. , Bai, S. , Diao, X. , Xiu, Y. , & Shen, Q. (2022). Effect of different processing methods on the millet polyphenols and their anti‐diabetic potential. Frontiers in Nutrition, 9, 101. 10.3389/fnut.2022.780499 PMC8873100 35223942

[fsn33659-bib-0106] Xiang, J. , Apea‐Bah, F. B. , Ndolo, V. U. , Katundu, M. C. , & Beta, T. (2019). Profile of phenolic compounds and antioxidant activity of finger millet varieties. Food Chemistry, 275, 361–368. 30724208 10.1016/j.foodchem.2018.09.120

[fsn33659-bib-0107] Yenasew, A. , & Urga, K. (2022). Effect of germination period on physiochemical properties of elite finger millet varieties. Cogent Food & Agriculture, 8(1), 2093045.

[fsn33659-bib-0108] Zhang, G. , Xu, Z. , Gao, Y. , Huang, X. , Zou, Y. , & Yang, T. (2015). Effects of germination on the nutritional properties, phenolic profiles, and antioxidant activities of buckwheat. Journal of Food Science, 80(5), H1111–H1119. 25858540 10.1111/1750-3841.12830

[fsn33659-bib-0109] Zvauya, R. , Mygochi, T. , & Parawira, W. (1997). Microbial and biochemical changes occurring during production of masvusvu and mangisi, traditional Zimbabwean beverages. Plant Foods for Human Nutrition, 51, 43–51. 9498693 10.1023/a:1007972428849

